# Point-of-Care Ultrasound is Having Its Moment

**DOI:** 10.24908/pocus.v5i1.14221

**Published:** 2020-07-06

**Authors:** Resa E Lewiss, Rachel B Liu, Robert Strony

**Affiliations:** 1 Thomas Jefferson University Philadelphia, PA; 2 Yale University School of Medicine New Haven, CT; 3 Geisinger Medical Center Danville, PA

**Keywords:** POCUS, Point-of-Care Ultrasound, COVID-19

## Letter:

Ultrasound performed at the point of care (POCUS) is having its moment. The COVID-19 pandemic has seemingly caused the acceleration of POCUS acceptance by hospital leaders, and POCUS examinations are increasingly performed by specialists outside of emergency medicine. What is driving this rapid culture change?

POCUS simply makes sense. This technology has been deemed essential to the safe management of patients amidst the COVID-19 pandemic 1. The clinical examination and imaging can be accomplished at the same time and by the same physician. The conditions of the pandemic have favored imaging modalities that can be rapidly deployed to the bedside, are quick to complete, involve easily disinfected instruments, and do not require multiple personnel. To date, there are no large outcome data on the use of POCUS for COVID-19, but published reports support that POCUS is time efficient and can accurately demonstrate clinical diagnoses, such as viral pneumonia 2, 3.

Cardiologists and radiologists have shown unprecedented support for POCUS during COVID-19. In fact, the pandemic has inspired non-emergency medicine specialties to write POCUS-related policies and guidelines. For example, the American Society of Echocardiography stated that clinicians caring for COVID-19 positive patients and those under investigation (PUI) should perform cardiac POCUS as a first-line imaging tool for cardiovascular findings. Doing so potentially replaces or delays the need for comprehensive transthoracic echocardiography or other imaging 4. This approach reduces the number of healthcare workers potentially exposed to an infected patient while improving efficiency. Similarly, an expert panel of radiologists from academic institutions in the United States and the American College of Radiology have discouraged the routine and first line use of computed tomography (CT) for diagnostic purposes in COVID-19 3, 4. Studies of COVID-19 pneumonia patients have shown similar test characteristics between lung POCUS and CT. There is growing support that lung POCUS is an accurate initial imaging tool for the early diagnosis of COVID-19 pneumonia, particularly in patients with flu-like symptoms 5, 6. The immediate imaging results and interpretation lead to more efficient care and better throughput from the ED to inpatient care areas. On the inpatient side, clinicians who use POCUS have less need for consultative services to perform diagnostic imaging and ultrasound-guided procedures, such as paracentesis or thoracentesis.

Machines designed for POCUS applications range in size from cart-based machines that are wheeled directly to the patient bedside, to handheld units which attach to smartphones and fit in a pocket. Machine screens, surfaces and transducers can be covered using translucent sheaths, and cleaned directly with viricidal wipes to be made ready for the next patient. On the other hand, obtaining a CT scan on a COVID-19 positive or PUI patient requires a more time-consuming cleaning and disinfection process, as well as adequate room ventilation and passive air exchange 7. The impact to workflow may be trivial at a hospital with multiple CT scanners, but significant to a hospital that has few. 

Hospital leaders working closely with Information Systems and Technology (IS&T) are prioritizing POCUS projects so that emergency and critical care physicians can digitally archive images to reduce duplication of imaging studies and enable image sharing with all consulting services. Archiving of POCUS studies has been historically difficult to achieve, but hospitals have increasingly engaged their IS&T departments during the pandemic. 

As POCUS use increases, there is a concomitant need for physician training. While dedicated proctored training is optimal, this may not be possible in a time of social distancing and course cancellations. Fortunately, novel teleguidance and augmented reality platforms have emerged on various handheld POCUS devices 7. Tele-ultrasound is being used by experienced faculty to deliver remote hands-on education for students and trainees including teaching proper scanning technique and demonstrating pathologic findings. POCUS teleguidance technologies may also aid frontline clinicians in remotely managing critical COVID-19 patients or discharged patients who are self-monitoring at home.

COVID-19 is rapidly changing the paradigm of how clinicians diagnose and manage patients at the bedside. Hospital leaders should embrace POCUS and ensure that proper equipment, training, and credentialing are readily available to frontline clinicians. 

## Conflicts of Interest

None declared.
